# Lycopene and *Garcinia cambogia* Induce White-to-Brown Adipose Differentiation: An Innovative Strategy to Curb Obesity

**DOI:** 10.3390/ph17080986

**Published:** 2024-07-25

**Authors:** Federica Mannino, Vincenzo Arcoraci, Giovanna Vermiglio, Davide Labellarte, Igor Pirrotta, Domenico Antonio Giorgi, Alessandro Scarfone, Alessandra Bitto, Letteria Minutoli, Mario Vaccaro, Mariarosaria Galeano, Giovanni Pallio, Natasha Irrera

**Affiliations:** 1 Department of Medicine and Surgery, University of Enna “Kore”, Contrada Santa Panasia, 94100 Enna, Italy; federica.mannino@unikore.it; 2 Department of Clinical and Experimental Medicine, University of Messina, Via C. Valeria, 98125 Messina, Italy; varcoraci@unime.it (V.A.); igor.pirrotta@unime.it (I.P.); domenicoantonio.giorgi@unime.it (D.A.G.); alessandro.scarfone@unime.it (A.S.); abitto@unime.it (A.B.); lminutoli@unime.it (L.M.); vaccaro@unime.it (M.V.); nirrera@unime.it (N.I.); 3 Department of Biomedical and Dental Sciences and Morphological and Functional Imaging, University of Messina, Via C. Valeria, 98125 Messina, Italy; giovanna.vermiglio1@unime.it (G.V.); davidelabellarte99@gmail.com (D.L.); mariarosaria.galeano@unime.it (M.G.)

**Keywords:** adipocyte differentiation, browning process, *Garcinia cambogia*, lycopene, obesity, uncoupling protein 1 (UCP1)

## Abstract

Obesity is considered one of the main risk factors for cardiovascular diseases. The browning process has been recently recognized as a promising anti-obesity therapy. Lycopene (LYC) and *Garcinia cambogia* fruit extract (GE) might be important resources for anti-obesity drugs; therefore, the aim of this study was to investigate the anti-obesity effects of LYC and GE on 3T3-L1 adipocytes and Zucker rats. Mouse 3T3-L1 pre-adipocytes were differentiated in mature adipocytes and then treated with LYC (0.5 μM), GE (30 mg/mL) or LYC + GE for 24 h. Moreover, male Zucker Crl:ZUC-Leprfa rats were randomly assigned to 5 groups of 10 animals to orally receive Vehicle (Ctrl), Orlistat (20 mg/kg), LYC (5 mg/kg), GE (1000 mg/kg) or LYC + GE for 28 days. LYC, GC extracts and even more LYC + GE stimulated the mRNA and protein expression of thermogenic genes *UCP1*, *CIDEA* and *DIO2*, significantly reduced lipid droplet size and increased lipid droplet number in adipocytes. UCP1 mRNA and protein expression was also increased in the visceral adipose tissue of the rats that received the dietary intake of LYC, GE and even more LYC + GE. Moreover, LYC + GE induced the reorganization of visceral fat depots that showed a great number of small adipocytes and a significant reduction in weight gain and food intake compared to the control group. The obtained results demonstrated that LYC + GE might be used as new approaches for obesity management in order to induce the browning process and achieve a metabolically active tissue instead of a tissue characterized by lipid depot accumulation.

## 1. Introduction

Obesity and metabolic syndrome (MetS) represent epidemic public health problems and are considered important risk factors for cardiovascular diseases, diabetes and cancer, also among children [1]. MetS is characterized by hypertension, dyslipidemia, glucose intolerance, as well as central obesity; visceral obesity may be responsible for insulin resistance, impaired lipolysis and increased free fatty acid (FFA) release; FFAs inhibit insulin effects and reduce glycogen synthesis, thus stimulating glucose production and lipogenesis [2]. Adipose tissue accumulation not only results in an increase in body fat with significant consequences on metabolism and increased risk of developing comorbidities related to obesity but also establishes a pro-inflammatory state due to adipokines release [3]. Obesity prevalence is projected to reach 20% or more in European countries by 2025, with dramatic consequences on healthcare systems [4]. In recent decades, Italy was a symbol of healthy lifestyle, with an obesity rate of only 8% of the population versus 30% in the US and 21% in the United Kingdom (UK), thanks to lifestyle and healthy diet [5]. In fact, obesity is a modifiable risk factor, and lifestyle intervention on physical activity and diet represents the first-line approach to reduce body weight [6]. Anti-obesity drugs together with lifestyle interventions are recommended for obese patients with a Body Mass Index (BMI) ≥ 30 kg/m^2^ or overweight patients (BMI ≥ 27 kg/m^2^) with at least one comorbidity, such as type 2 diabetes, cardiovascular disease and osteoarthritis [7]. The main mechanism of action of the approved anti-obesity drugs, such as GLP-1 agonists, is based on the modulation of food intake regulation, hunger/food reward reduction and satiation, whereas orlistat acts with a different mechanism, by inactivating gastric and pancreatic lipases, with the consequent excretion of up to 30–35% of the ingested fat [8,9]. The extracts of natural origin would seem to be an interesting source for the development of anti-obesity products and, in particular, for metabolic syndrome and obesity prevention [10]. In this context, *Garcinia cambogia* fruit extract (GE) and the isolated hydroxycitric acid (HCA) showed anti-diabetic, hypolipidemic and anti-obesity effects, as well as lycopene (LYC), thanks to its anti-oxidant/anti-inflammatory properties [11,12]. Recent studies demonstrated that LYC might also exert anti-obesity effects through the activation of the trans-differentiation of the white adipose tissue (WAT) into the brown adipose tissue (BAT), the so-called browning process, thus inducing thermogenic gene expression through the regulation of PPARγ [13]. In particular, WAT is composed of lipid-storing cells, with a single lipid droplet, characterized by low mitochondrial density, whereas BATs show multilocular adipocytes which contain many small lipid droplets and a large number of oxidative mitochondria that oxidize glucose and lipids in order to produce heat, making a metabolically active tissue.

The scientific evidence described so far indicates that both HCA and LYC may serve as novel natural products for the development of anti-obesity drugs and for the prevention of obesity and MetS. Therefore, the present study aimed at investigating the anti-obesity effects of a combination of LYC and GE in both in vitro and in vivo experimental models of obesity. 

## 2. Results

### 2.1. LYC and GE Do Not Affect Cell Viability

**Figure 1:**
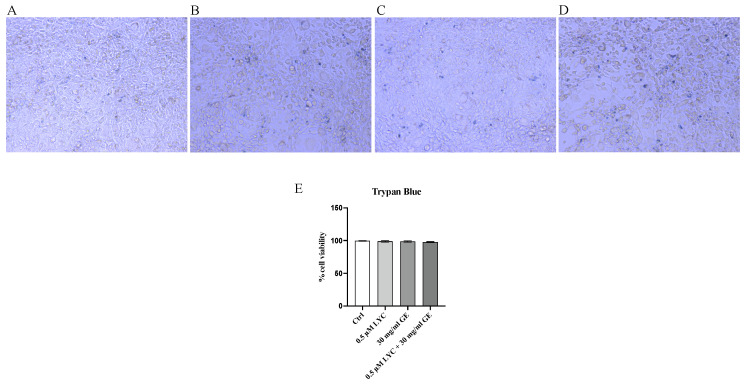
Panels (**A**–**D**) show cytotoxicity evaluated by Trypan Blue exclusion test in 3T3-L1 cells; Ctrl (**A**), LYC (**B**), GE (**C**) and LYC + GE (**D**). Panel (**E**) summarizes the results of the Trypan Blue exclusion test; all images were captured at 10× magnification. Values are expressed as the mean and SD of three experiment.

Trypan Blue exclusion test was performed to evaluate 3T3-L1 cell viability following treatment with LYC, GE and LYC + GE. The images obtained by the trypan blue exclusion test showed that 3T3-L1 cell viability was not influenced by the treatment with LYC (0.5 μM) or GE (30 mg/mL) alone and in combination, thus demonstrating that the used compounds did not have cytotoxic effects on 3T3-L1 cells (Figure 1A–E).

**Figure 2:**
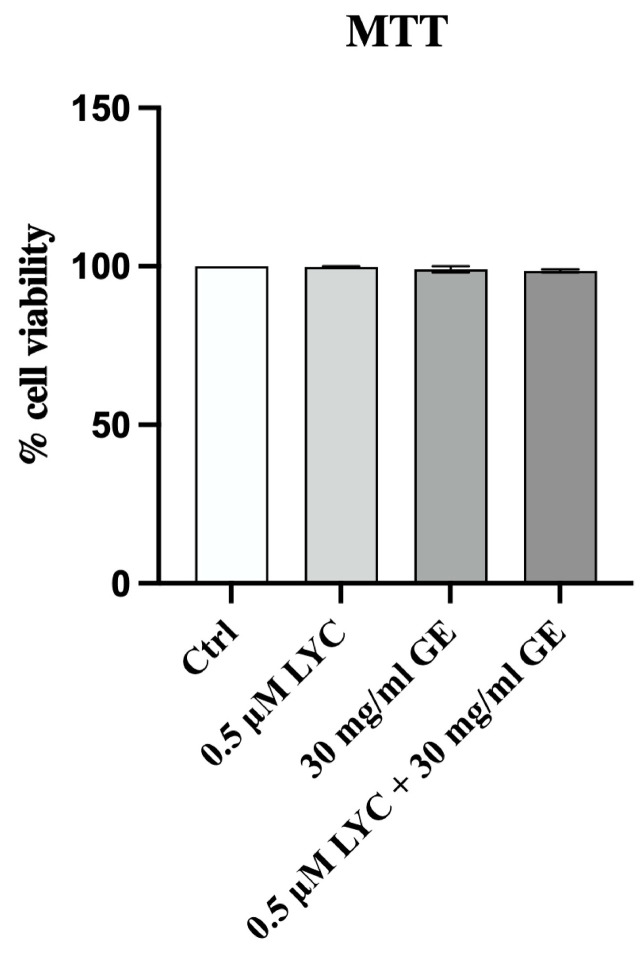
Cell viability evaluated in 3T3-L1 cells treated with LYC, GE and LYC + GE at 24 h by MTT assay. Values are expressed as the mean and SD.

In order to confirm that LYC, GE and their combination were not cytotoxic, MTT assay was performed at 24 h. The graph shows that LYC, GE and LYC + GE did not affect cell viability at 24 h, thus demonstrating that the tested compounds did not have a cytotoxic effect on 3T3-L1 cell line (Figure 2).

### 2.2. LYC and GE Stimulate Trans-Differentiation from White to Brown Adipocytes

**Figure 3:**
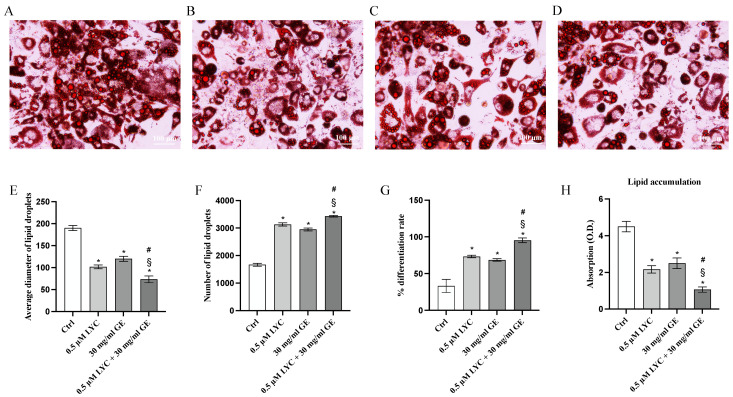
The panels show differentiated adipocytes stained with ORO staining of Ctrl (**A**), LYC (**B**), GE (**C**) and LYC + GE (**D**). The graphs represent average diameter of lipid droplets (**E**), number of lipid droplets (**F**), % differentiation rate (**G**) and ORO absorption (**H**) obtained from each group. Values are expressed as the mean and SD of three experiment; * *p* < 0.05 vs. Ctrl; # *p* < 0.05 vs. LYC; § *p* < 0.05 vs. GE.

ORO staining was performed to evaluate whether LYC and GE, alone or in combination, were able to promote white to brown adipocyte trans-differentiation. ORO staining demonstrated that LYC and GE significantly reduced the diameter of lipid droplets, and consequently lipid droplets size compared to untreated cells; the coincubation of LYC and GE showed a greater effect than that observed when LYC and GE were used alone, as demonstrated in Figure 3E. Moreover, lipid droplet number and differentiation rate were markedly increased following treatment with LYC and GE, especially when the natural compounds were used together, as confirmed by ORO absorption, thus demonstrating that LYC, GE and even more their combination were able to significantly reduce lipid accumulation compared to control cells (Figure 3F–H).

### 2.3. LYC and GE Stimulate the Browning Process

**Figure 4:**
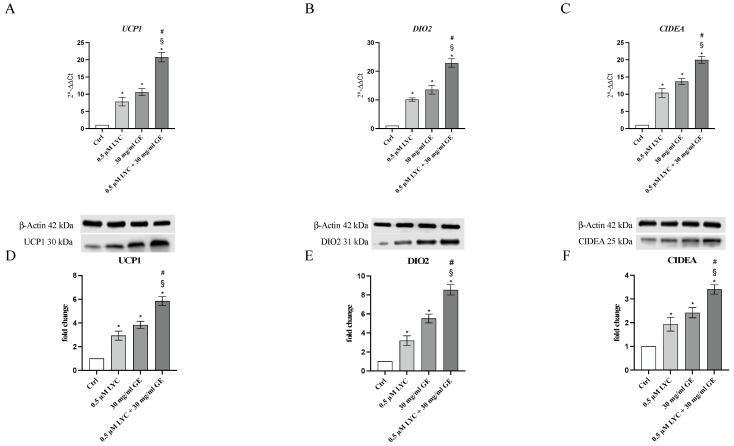
The graphs represent UCP1 (**A**), DIO2 (**B**) and CIDEA (**C**) mRNA expression (RTqPCR analysis) and UCP1 (**D**), DIO2 (**E**) and CIDEA (**F**) protein expression (western blot analysis) obtained from 3T3-L1 cells. Values are expressed as the mean and SD; * *p* < 0.05 vs. Ctrl; # *p* < 0.05 vs. LYC; § *p* < 0.05 vs. GE.

The mRNA expression of UCP1, DIO2 and CIDEA was evaluated to study whether LYC and GE as well as their combination were able to induce the browning process through the stimulation of the specific markers. qPCR analysis highlighted that the treatment with LYC or GE markedly increased the expression of genes involved in the browning process compared to untreated cells, with a greater effect following LYC + GE treatment (Figure 4A–C). UCP1, DIO2 and CIDEA protein expression was also investigated to confirm LYC and GE effects on the browning process: all browning markers were significantly increased following LYC or GE treatment and even more after the incubation with the association (Figure 4D–F).

### 2.4. LYC and GE Effects on Zucker Rats Body Weight Gain and Food Intake

**Figure 5:**
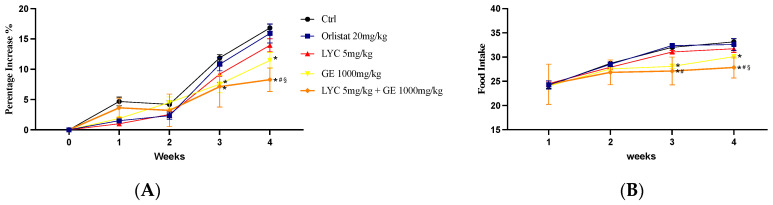
The graphs show the body weight gain percentage (**A**) and the food intake (**B**) from day 0 until the end of the experiment. Values were obtained from 10 animals per group and are expressed as means and SD; * *p* < 0.05 vs. Ctrl; # *p* < 0.05 vs. LYC; § *p* < 0.05 vs. GE.

Body weight gain and food intake could be essential parameters to evaluate the effects of LYC and GE, alone or in combination. Control group and LYC-treated Zucker rats showed a similar percentage of weight gain throughout the experiment (28 days). Also, the group that received the oral administration of orlistat showed a comparable percentage increase in weight gain. Thus, no significant difference was observed at all time points. On the other hand, GE-treated animals and especially LYC + GE-treated rats showed a percentage of body weight gain significantly reduced compared to Ctrl after 21 days and even more at the end of the experiment (Figure 5A). Food intake was unchanged in orlistat and LYC-treated groups compared to Ctrl throughout the experimental period. Instead, GE-treated animals and even more LYC + GE-treated rats showed a significant reduction of food intake compared to Ctrl after 21 days and especially at the end of the experiment.

### 2.5. LYC and GE Stimulate the In Vivo Trans-Differentiation from White to Brown Adipocytes

**Figure 6:**
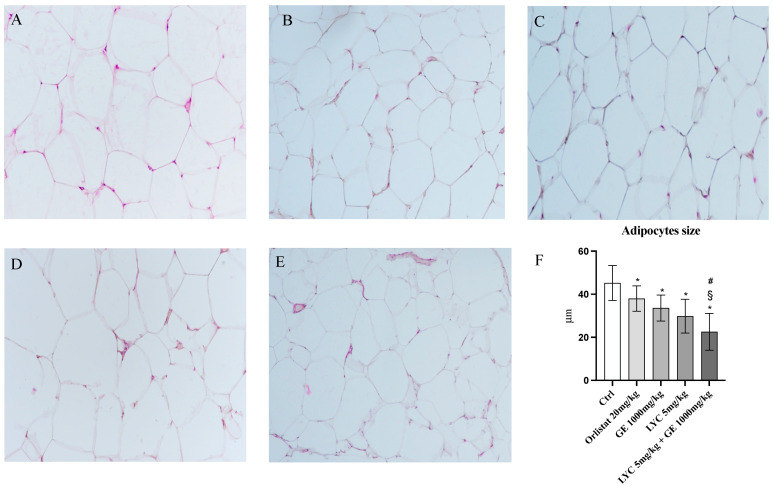
Panel of H&E sections of visceral fat depots. Ctrl animals (**A**) exhibit the typical structure of large unilocular adipocytes of the same size; orlistat (**B**) and GE (**C**) treated animals show the presence of unilocular adipocytes of different size; LYC-treated animals (**D**) show the presence of large unilocular adipocytes and small unilocular adipocytes; LYC + GE treated rats (**E**) show a visceral adipose tissue organized by adipocytes of different dimension with an inhomogeneous aspect and small lipid droplets. Panel (**F**) shows the quantitative difference in adipocytes size; the values were obtained from 10 slides for each group and the diameter of 10 adipocytes randomly selected for each slide was measured; all images were captured at 20× magnification. The data are expressed as means and SD. * *p* < 0.05 vs. Ctrl; # *p* < 0.05 vs. LYC; § *p* < 0.05 vs. GE.

**Figure 7:**
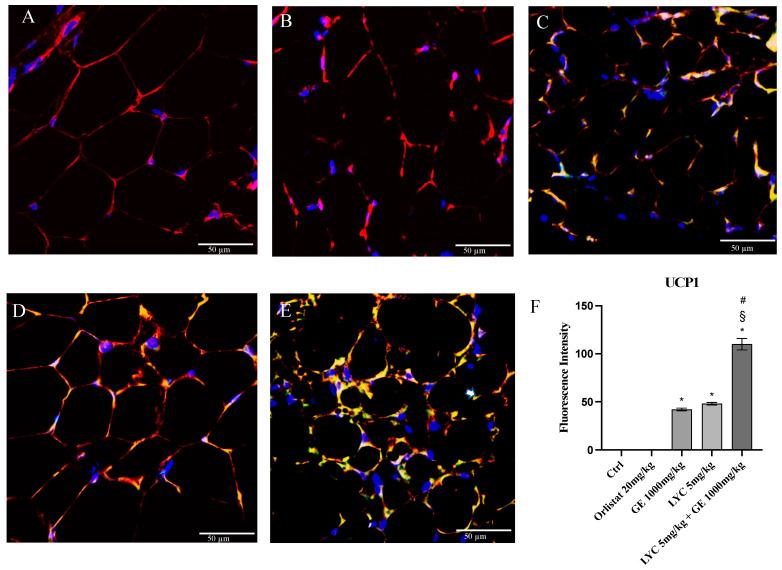
Immunofluorescence reactions with anti-UCP1 (green channel) and anti-PGC1α (red channel) in visceral fat depots of Zucker rats. Ctrl animals (**A**) show large unilocular adipocytes positive for PGC1α but negative for UCP1; orlistat (**B**) treated animals show visceral fat depots with unilocular adipocytes positive for PGC1α only but negative for UCP1; GE (**C**) and LYC (**D**) treated animals show unilocular adipocytes with different size positive for both PGC1α and UCP1. LYC + GE (**E**) treated animals show visceral fat depots with great number of small adipocytes positive for both PGC1α and UCP1; many adipocytes are reorganizing their structure with numerous lipid droplets delimited by membrane (arrow). Panel (**F**) shows the fluorescent intensity of UCP1 obtained by the confocal laser. Values were obtained from 10 animals per group and are expressed as means and SD; * *p* < 0.05 vs. Ctrl; # *p* < 0.05 vs. LYC; § *p* < 0.05 vs. GE.

**Figure 8:**
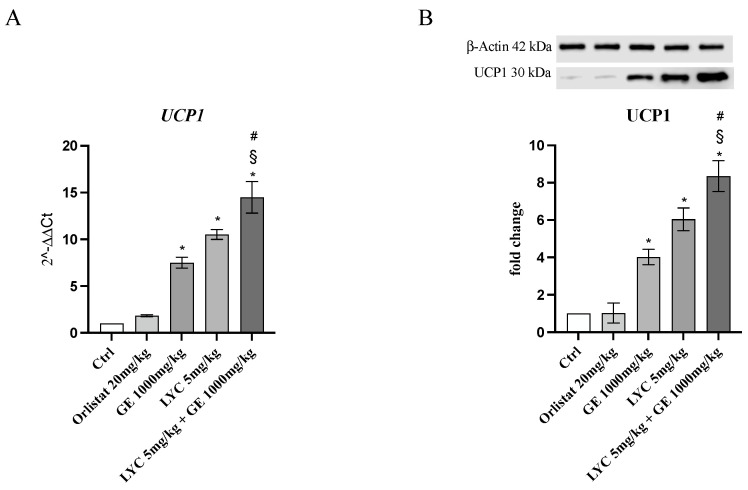
The graphs represent UCP1 mRNA expression (**A**) and UCP1 protein expression (**B**) obtained from visceral fat depots of Zucker rats. Values are expressed as the mean and SD; * *p* < 0.05 vs. Ctrl; # *p* < 0.05 vs. LYC; § *p* < 0.05 vs. GE.

H&E staining was performed to evaluate adipocytes size of visceral fat depots following treatment with LYC and GE, alone or in combination. As shown in Figure 6A, untreated animals (Ctrl) exhibit the typical structure of large unilocular adipocytes of the same size; Zucker rats treated with orlistat and GE still showed unilocular adipocytes although with different size compared to controls (Figure 6B,C). Large unilocular adipocytes and small unilocular adipocytes were observed in LYC-treated animals (Figure 6D). Moreover, in the group of animals treated with LYC + GE combination an inhomogeneous shape with adipocytes of different dimensions and visceral fat depots with small lipid droplets may be detected (Figure 6E). The differences in adipocyte size between the groups were summarized in the Figure 6F. To confirm LYC and GE effects on trans-differentiation from WAT to BAT, immunofluorescence analysis was performed in visceral fat depots to evaluate the expression of UCP-1 and PGC1α, as key regulators of the browning process. Also following immunofluorescence, large unilocular adipocytes positive for PGC1α but negative for UCP1 may be observed in controls (Figure 7A); orlistat treated animals showed visceral fat depots with unilocular adipocytes positive only for PGC1α but negative for UCP1 (Figure 7B); many unilocular adipocytes with different size positive for both PGC1α and UCP1 may be detected in the GE and LYC-treated groups (Figure 7C,D). In contrast, following LYC + GE treatment, visceral fat depots showed a great number of small adipocytes positive for both PGC1α and UCP1 (Figure 7E); moreover, many adipocytes seem to start a reorganization of their structure with numerous lipid droplets. In order to confirm the data obtained by the immunofluorescence analysis, UCP1 mRNA expression as well as protein expression were also evaluated by RTqPCR and western blot analyses. The treatment with LYC or GE and even more the administration of their combination markedly increased UCP1 mRNA and protein expression compared to untreated animals; in contrast, orlistat administration did not stimulate UCP1 expression and any effect was observed compared to Ctrl (Figure 8A,B).

## 3. Discussion

Natural products represent a tempting resource for the discovery of drug candidates to treat different diseases and, in particular, natural extracts such as LYC derived from tomato and HCA obtained from *Garcinia cambogia* fruit might be useful for fat metabolism regulation and body weight reduction in order to treat obesity [14,15,16]. In the last few years, the browning process, which leads to the trans-differentiation of WAT into BAT, has been recognized as a possible new strategy to treat obesity and other metabolic-related diseases. WAT is mainly considered a storage depot for lipids, whereas BAT is involved in nonshivering thermogenesis [17]. Adipocytes may differentiate from white adipocytes to brown-like adipocytes, beige or brite adipocytes, in response to different stimuli, thus acquiring a thermogenic activity [18,19]. Beige cells although resemble white adipocytes may express thermogenic genes and show an increased mitochondrial activity, thus reducing the risk of obesity. Therefore, both BAT and beige adipocyte stimulation could potentially represent a therapeutic approach, together with the involvement of different signaling pathways. In this context, UCP1 is one of the main effectors of the adaptive thermogenesis in BAT that induces movement of protons back into the mitochondrial matrix, thus activating respiratory pathways and FAs [20]. UCP1 may be induced by cold exposure with the consequent activation of BAT thermogenesis [21]; however, this application is uncomfortable and may be related to an increase in cardiovascular risk in patients [22]. Also, Mirabegron, a β3-AR agonist, as well as Troglitazone, may stimulate UCP1 expression and consequently browning process, although their use shows different limitation in the application for obesity, including a reduced effectiveness and the appearance of side effects [23,24]. Dietary products may stimulate thermogenic genes in WAT, such as UCP1, thus inducing browning process [25,26]. In accordance with previous scientific evidences, we observed that LYC induced not only the expression of UCP1 but also of CIDEA and DIO2, representing the signature of beige and brown selective genes, probably as a consequence of PPARα and PPARγ modulation [13,27]. In particular, CIDEA plays a crucial role in the development of adipose tissue wasting through browning activation and lipolysis increase, by regulating UCP1 for britening and thermogenesis together with PGC-1α [28]. Moreover, also GE treatment increased the gene and protein expression of these browning markers; these effects were mediated by HCA (the main component of GE) that is able to decrease the production of malonyl-CoA in hepatocytes by inhibition of ATP: citrate lyase and activation of CPT 1 stimulating lipid oxidation and thermogenesis [29]. Furthermore, a greater effect in the induction of browning was observed following the treatment with the combination of LYC and GE compared to control cells: all treatments induced the browning process and significantly stimulated UCP1, CIDEA and DIO2 gene and protein expression. These molecular results are supported by the cell morphology highlighted by the ORO staining: the trans-differentiation process was evident following the treatment with LYC or GE and especially when LYC and GE were used in combination. Moreover, LYC, GE and even more LYC + GE treatment significantly reduced cell volume and lipid droplet size as well as increased lipid droplets number compared to untreated adipocytes, further demonstrating browning induction. In order to support the obtained in vitro data, Zucker rats were used as a genetic model of obesity caused by the mutation (fa) in the gene encoding the receptor of leptin, which leads to hyperphagia and excessive adiposity. We observed that UCP1 gene and protein expression was increased in the visceral adipose tissue of the animals that received the dietary intake of LYC, GE and even more their combination compared to untreated animals. Moreover, areas of small adipocytes were detected in all treated groups as well as the presence of smaller lipid droplets in the LYC + GE treated animals, thus demonstrating that these natural compounds were able to stimulate the browning of WAT. In contrast, the visceral adipose tissue obtained from the orlistat-treated animals still showed the phenotype of WAT with unilocular adipocytes positive for PGC1α only but negative for UCP1; in fact, its mechanism of action is mainly related to fat absorption inhibition and fat mass reduction, with no effect on browning [30]. Fat accumulation is also dependent on appetite which regulates body weight and is usually controlled by the appetite centers and hypothalamus, as well as hormones released by the gut and periphery [31]. In this study, an increase in body weight gain percentage was observed throughout the experiment; however, GE-treated animals and even more LYC + GE-treated rats showed a significant reduction of body weight gain and food intake parameters compared to untreated animals following 21 days of treatment and especially at the end of the experiment. These results were in accordance with a previous paper that demonstrated the positive effect of GE in reducing food-intake and body weight gain in Zucker rats compared to controls [32]. In contrast, a randomized control study demonstrated no significant change on weight reduction following the use of HCA supplementation even due to different factors, such as duration and dose of exposure [33]. Moreover, the data on weight are still controversial for carotenoids that on one hand may contribute to weight and BMI reduction in overweight and obese patients but other studies did not report significant effects in Zucker fatty rats and in overweight patients [34,35,36,37,38,39]. Therefore, we hypothesized that our results may be the consequence of (i) the duration of treatment (28 days of dietary intake are not enough for body weight reduction consequent to browning activation) and (ii) the animal model used, since the absence of leptin would reduce energy expenditure by contributing to weight gain. However, our results are quite fascinating since a significant reduction of the weight gain percentage was observed following LYC + GE administration.

## 4. Material and Methods

### 4.1. 3T3-L1 Pre-Adipocytes Cell Differentiation and Treatments

Mouse 3T3-L1 pre-adipocytes were cultured in Dulbecco’s Modified Eagle’s Medium (DMEM; ATCC, Manassas, VA, USA) high glucose supplemented with heat-inactivated Fetal Bovin Serum (FBS; ATCC, Manassas, VA, USA) (10%), 100 U/mL penicillin and 100 μg/mL streptomycin (ATCC, Manassas, VA, USA) at 37 °C in a humidified incubator with 5% of CO_2_. Once reaching 70–90% confluence, pre-adipocytes were differentiated in mature adipocytes by replacing the culture medium with a differentiation medium (MDI), composed of DMEM supplemented with 10% FBS, 0.5 mM 3-isobutyl-1-methylaxanthine (IBMX), 1.0 µM dexamethasone and 1.5 µg/mL insulin (Sigma Aldrich, St. Louis, MO, USA). After 48 h of induction, differentiation medium was replaced with a growth medium containing DMEM supplemented with 10% FBS and 1.5 µg/mL insulin; the growth medium was replaced every 2 days until day 14 [40].

Following 14 days of differentiation, mature adipocytes were treated with LYC (0.5 μM; 98% purity; Hering srl, Modica, Italy) or GE (30 mg/mL; 70% HCA with minor components of: Oxy-guttiferone I, K, K2, M; Guttiferone I, N, J, K, M; Hering srl, Modica, Italy) or the combination of LYC and GE (0.5 μM and 30 mg/mL respectively) for 24 h.

### 4.2. Trypan Blue Exclusion Test of Cell Viability

Trypan Blue test was performed to evaluate the number of viable cells following LYC, GE and LYC + GE treatment. In detail, cells were centrifuged for 5 min at 1200 rpm and resuspended in 1 mL of sterile PBS; cell suspension were diluted mixing 1 part of 0.4% trypan blue and 1 part of cell suspension. Mixture was incubated for 3 min at room temperature; 10 μL was loaded in a hemacytometer and immediately observed under a microscope to count the number of cells stained in blue and unstained. To calculate the total number of viable cells per ml of aliquot, the total number of viable cells was multiplied by 2 (the dilution factor for trypan blue) and the percentage was estimated as follows [41]:

$$\mathrm{viable}\;\mathrm{cells}\;\left(\%\right)=\frac{\mathrm{total}\;\mathrm{number}\;\mathrm{of}\;\mathrm{viable}\;\mathrm{cells}\;\mathrm{per}\;\mathrm{mL}\;\mathrm{of}\;\mathrm{aliquot}\;}{\mathrm{total}\;\mathrm{number}\;\mathrm{of}\;\mathrm{cells}\;\mathrm{per}\;\mathrm{mL}\;\mathrm{of}\;\mathrm{aliquot}}\times 100$$

### 4.3. MTT Assay

Cell viability was evaluated by MTT assay following treatment with LYC, GE and LYC + GE. In particular, 3T3-L1 cells were cultured in a 96-well plate with a density of 1 × 10^5^ cell/well for 24 h in order to test the cytotoxic effects of the studied compounds. The tetrazolium dye MTT 3-(4,5-dimethylthiazol-2-yl)-2,5-diphenyltetrazolium bromide (Sigma Aldrich, St. Louis, MO, USA), was dissolved in sterile filtered PBS, and 20 μL of the mixture were added into each well 5 h before the end of incubation (24 h). Medium was removed and the insoluble formazan crystals were dissolved with dimethyl sulfoxide (DMSO; 200 μL/well; Sigma Aldrich, St. Louis, MO, USA) following 5 h. The difference between the values obtained at 540 and 620 nm of absorbance was used to calculate the average of replicates and to evaluate cytotoxicity. Results were expressed as % of cell viability compared to untreated cells and reported as means and SD [42].

### 4.4. Oil Red O Lipid Staining and Lipid Accumulation

After LYC, GE and LYC + GE treatment, cells were washed twice with sterile PBS, fixed with formaldehyde and stained with the Oil Red O (ORO) solution (40% water and 60% ORO stock solution; Sigma Aldrich, St. Louis, MO, USA), for 50–60 min; in a further set of plates, lipid accumulation was also measured as previously reported [43]. To quantify the lipid accumulation of each group, we used 100% isopropanol to dissolve the ORO and measured the absorbance at 490 nm. ImageJ 1.53e software for Windows (Softonic, Barcelona, Spain) was used to measure the number of lipid droplets and the average diameter of lipid droplets calculated as total area/number of droplets. Moreover, the differentiation rate was calculated as the number of lipid droplets containing adipocytes per total number of cells. The images are the representative of a set of three for each condition.

### 4.5. Real-Time Quantitative PCR Amplification

Total mRNA was isolated from mature adipocytes and visceral adipose tissue with Trizol LS reagent (Thermo Fisher Scientific, Waltham, MA, USA), quantified by using NanoDrop spectrophotometer (Thermo Fisher Scientific, Waltham, MA, USA) and retrotranscribed by using Superscript IV master mix (Thermo Fisher Scientific, Waltham, MA, USA). cDNA (1 μL) was added to the BrightGreen qPCR Master Mix (Applied Biological Materials Inc., Richmond, BC, Canada) in a total volume of 20 µL per well to evaluate the gene expression of *UCP1*, *DIO2* and *CIDEA*. The final primer concentration selected to perform the analysis was 10 μM. Samples were run in duplicate and qPCR reaction was monitored by QuantStudio 6 Flex Real-Time PCR System (Applied Biosystems, Foster City, CA, USA); the results were quantified by using the 2^ΔΔCt^ method by using *GAPDH* as housekeeping gene [44,45,46]. The primers for targets and housekeeping genes are listed in Table 1.

### 4.6. Western Blot

Total protein content was extracted from 3T3-L1 cells and visceral adipose tissue by using RIPA buffer with proteinase inhibitors; cell supernatants were used for quantification with Bradford method following centrifugation. Proteins (30 μg) were run by electrophoresis on a 10% SDS polyacrylamide gel and transferred to PVDF membranes (Amersham, Little Chalfont, UK) at 200 mA for 1 h. The obtained membranes were incubated with non-fat dry milk (5%), washed 3 times with TBS/0.1% Tween buffer and then incubated with primary antibodies for UCP1 (1:500, Cell Signaling, Danvers, MA, USA), DIO2 and CIDEA (1:1000, Abcam, Cambridge, UK) diluted in TBS-0.1% Tween, overnight at 4 °C. The day after, a secondary peroxidase-conjugated goat anti-rabbit antibody (KPL, Gaithersburg, MD, USA) was used for 1 h at room temperature. Images were obtained and quantified by a scanning densitometry with a bio-image analysis system (C-DiGit, Li-cor, Lincoln, NE, USA). Data were expressed as fold change compared to control and β-actin (Cell Signaling, Danvers, MA, USA) was used as loading control: the images showed in the figure were the most representative of three replicates [47].

### 4.7. Experimental In Vivo Model

Male Zucker Crl:ZUC-Leprfa rats (*n* = 50; Charles River, Calco, Italy) of 12 weeks of age were used in this study. Animals were housed in the Animal Facility of the Department of Clinical and Experimental Medicine of the University of Messina and provided with a standard diet ad libitum as well as free access to water. Rats were daily monitored and maintained in 12 h light/darkness cycle and at 24 °C. All the experimental procedures complied with the ARRIVE guidelines [48], with the Italian Guidelines for Care and Use of Laboratory Animals (D.L.116/92) and with the European Directive (2010/63/EU).

For the experimental procedure, Zucker rats were randomly assigned to 5 groups of 10 animals: Controls (Sunflowers oil, 200 μL), Orlistat (20 mg/kg), LYC (5 mg/kg; Hering srl Modica, Italy), GE (1000 mg/kg; Hering srl Modica, Italy) and LYC + GE (5 mg/kg and 1000 mg/kg respectively). Rats were daily administered by oral gavage for 28 days and then anesthetized with ketamine (100 mg/kg) and xylazine (10 mg/kg) to be killed; visceral fat was collected for histological analysis. Animals were weighed once a week between 9:00 and 10:00 a.m. throughout the experiment. Body weight was recorded every week and weight gain was evaluated as percentage of increase. Food intake was evaluated every day between 9:00 and 10:00 a.m. and expressed in g/die as mean and standard deviation (SD) of multiple measurements for each week.

### 4.8. Morphological Analysis

Visceral adipose tissue was fixed in paraformaldehyde (4%), buffered in 0.1 M sodium cacodylate (pH 7.4) at room temperature for 4 h. After rinses in phosphate buffer 0.13 mol/L, pH 7.3, the specimens were dehydrated in ethanol and embedded in paraffin. Five-µm-thick sections were obtained by using a LEICA microtome and stained with hematoxylin and eosin (H&E) and processed for immunofluorescence analysis. The adipocytes size was evaluated in slides of visceral adipose tissue stained with haematoxylin-eosin by using ImageJ 1.53e software for Windows (Softonic, Barcelona, Spain) [49]. In detail, 10 slides were selected for each group to measure the diameter of 10 adipocytes for each slide, randomly selected. For the adipocytes that were not perfectly spherical, the diameter of the minor axis was calculated.

### 4.9. Immunofluorescence Analysis

Double localization reactions have been performed by using rabbit anti-UCP1 and goat anti-PGC1α (Peroxisome proliferator-activated receptor gamma coactivator 1-alpha) antibodies. Primary antibodies were incubated for 2 h at room temperature and after several rinses in PBS, FITC anti-rabbit (1:100 dilution; Jackson Immuno-Research Lab., West Grove, PA, USA) and Texas-Red-conjugated IgG anti-goat (1:100 dilution; Jackson Immuno-Research Lab., West Grove, PA, USA) were used, respectively. The fluorochrome was applied for 1 h at room temperature and, after numerous rinses in PBS, sections were incubated with DAPI diluted 1:1000 in PBS (Sigma Aldrich, St. Louis, MO, USA), for 10 min at room temperature, in order to label nuclei. Slides were then washed in PBS and sealed with the mounting medium. Samples were observed with a Zeiss LSM 510 confocal microscope equipped with Argon laser (458 nm and 488 nm) and two HeNe laser (543 nm and 633 nm). All images were digitized at a resolution of 8 bits into an array of 2048 × 2048 pixels. Optical sections of fluorescence specimens were obtained at 488 nm, at 62/s scanning shipped with up to 8 repetitions on average. The pinhole was set for optimal resolution; contrast and brightness were established by examining the most brightly labeled pixels and choosing settings that allowed clear visualization of structural details while keeping the highest pixel intensities near 200. The same parameters have been used for all the observations. Digital images were cropped and figure montages prepared using Adobe Photoshop 7.0 [50].

### 4.10. Statistical Analysis

The data are expressed as mean ± SD. All in vitro experiments were performed in triplicate. The differences between the experimental groups were analyzed by using the one-way and two-way ANOVA approaches with the Tukey’s post-test for intergroup comparisons. A *p* value < 0.05 was considered significant, and graphs were prepared by using the GraphPad Prism software (Version 8.0 for macOS, San Diego, CA, USA).

## 5. Conclusions

The present study demonstrated that dietary natural products, such as LYC and GE, may be used as new approaches for obesity management in order to induce browning process and achieve a metabolically active tissue instead of a tissue characterized by lipid depot accumulation. Nevertheless, the present study shows some limitations; first of all, pre-treated groups were not included to demonstrate the possible preventive effects of LYC, GE and LYC + GE, that could be used as dietary supplementation to prevent overweight and obesity-related conditions nor a group treated with mirabregon which is considered one of the gold-standard treatments for browning induction. However, our main goal was to assess whether LYC, GE and in particular LYC + GE could be considered as therapeutic option in association with a balanced diet and potentially with the already approved drugs. Moreover, considering that obesity could also be a gender-related condition [51], the evaluation of LYC, GE, LYC + GE effects in both sexes could be interesting and could provide significant additional information; however, the efficacy of LYC + GE supplementation was demonstrated in the present paper, indicating that the combination of these extracts could be used as supportive therapy for the management of obesity due to their potential in stimulating browning process although our hypothesis needs to be further proven with additional studies and in a clinical setting.

## Figures and Tables

**Table 1:** Primers list for qPCR analysis.

Gene	Species	Sequence (5′→3′)	Length	Tm	GC%
*GAPDH*	Mouse	F: AAGAGGGATGCTGCCCTTAC	20	59.45	55
		R: CTCGTGGTTCACACCCATCA	20	59.97	55
*UCP1*	Mouse	F: CACGGGGACCTACAATGCTT	20	60.04	55
		R: ACAGTAAATGGCAGGGGACG	20	60.04	55
*DIO2*	Mouse	F: AGAAGTCCGAAGTTGGCTGG	20	59.96	55
		R: TCACACTTGGGAATTCGGGG	20	59.96	55
*CIDEA*	Mouse	F: AGAAGGTCCTACTGACCCCC	20	59.96	60
		R: ACCCGGTGTCCATTTCTGTC	20	59.96	55
*GAPDH*	Rat	F: TGTGAACGGGTGAGTTCCAG	20	59.89	55
		R: TACTTCGGCCACCCTATCCA	20	60.03	55
*UCP1*	Rat	F: ATCAAACCCCGCTACACTGG	20	60.04	55
		R: GCATAGGAGCCCAGCATAGG	20	60.04	60

## Data Availability

The raw data supporting the conclusions of this article will be made available by the authors on request.

## Abbreviations

Brown adipose tissue (BAT); Body Mass Index (BMI); Carnitine palmitoyltransferase I (CPT1); Cell Death Inducing DFFA Like Effector A (CIDEA); Iodothyronine deiodinase 2 (DIO2); Dulbecco’s Modified Eagle’s Medium (DMEM); Fetal Bovin Serum (FBS); free fatty acids (FFAs); *Garcinia cambogia* fruit extract (GE); hydroxycitric acid (HCA); lycopene (LYC); metabolic syndrome (MetS); Oil Red O (ORO); white adipose tissue (WAT); 3-isobutyl-1-methylaxanthine (IBMX); 3-(4,5-dimethylthiazol-2-yl)-2,5-diphenyltetrazolium bromide (MTT).
